# Transcutaneous Electrical Spinal Cord Stimulation Increased Target-Specific Muscle Strength and Locomotion in Chronic Spinal Cord Injury

**DOI:** 10.3390/brainsci14070640

**Published:** 2024-06-26

**Authors:** Niraj Singh Tharu, Arnold Yu Lok Wong, Yong-Ping Zheng

**Affiliations:** 1 Department of Biomedical Engineering, The Hong Kong Polytechnic University, Hong Kong SAR, China; singh.tharu@connect.polyu.hk; 2 Department of Rehabilitation Sciences, The Hong Kong Polytechnic University, Hong Kong SAR, China; arnold.wong@polyu.edu.hk; 3 Research Institute for Smart Ageing, The Hong Kong Polytechnic University, Hong Kong SAR, China

**Keywords:** transcutaneous electrical spinal cord stimulation, activity-based therapy, lower limb, muscle strength, locomotion, spinal cord injury

## Abstract

Background: The recovery of locomotion is greatly prioritized, and neuromodulation has been emerging as a promising approach in recent times. Study design: Single-subject research design. Settings: A laboratory at The Hong Kong Polytechnic University. Objectives: To investigate the effects of augmenting activity-based therapy (ABT) to transcutaneous electrical spinal cord stimulation (TSCS) on enhancing specific lower limb muscle strength and improving locomotor ability in an individual with chronic incomplete spinal cord injury (iSCI). Methods: An individual with iSCI underwent two phases of treatment, ABT alone followed by combined ABT+TSCS, each for a period of 10 weeks. The TSCS stimulated T10-T11 and T12-L1 segments with a frequency of 30 Hz at an intensity between 105 mA and 130 mA. Manual muscle testing, 6 min walk test (6MWT), and surface electromyography (EMG) responses of specific lower limb muscles were measured. Additionally, spasticity and sensorimotor examinations were conducted every two weeks, while pain tolerance was recorded after each treatment session. Results: After the ABT+TSCS treatment, there was an increase in overall muscle strength grading (from 1.8 ± 0.3 to 2.2 ± 0.6 out of 5.0). The 6MWT showed a greater increase in walking distance (3.5 m to 10 m) after combined treatment than ABT alone. In addition, the EMG response of the anterior rectus femoris, biceps femoris, medial gastrocnemius, and tibialis anterior after ABT+TSCS increased more than after ABT alone. The spasticity grade was reduced (from 0.8 ± 0.7 to 0.5 ± 0.6) whereas the average lower limb motor score increased from 17 to 23 points. No adverse effects were reported. Conclusions: ABT+TSCS increased the target-specific lower limb muscle strength and walking ability more than ABT alone in an individual with chronic iSCI.

## 1. Introduction

Spinal cord injury (SCI) leads to sensory, motor, and autonomic dysfunctions [1,2], resulting in irreversible disability [3,4]. Paralysis is one of the most common serious consequences of SCI that occurs immediately and may last for the rest of a person’s life [5]. People with incomplete SCI (iSCI) comprise approximately 51% of all SCI cases [6]. They experience various levels of paralysis, which impairs their mobility, transfer, and self-care, causing functional restrictions [6,7]. The weakness of a specific muscle is a typical motor symptom of iSCI, especially for individuals having some voluntary controls of their legs within limited ranges of motion [8]. People with iSCI are expected to have a 50% chance to restore their walking ability [9]. Therefore, they consider improving locomotion as their topmost priority [10].

When an individual with SCI progresses to the chronic phase, spontaneous recovery is unlikely [11]. They are often left with significant motor deficits despite receiving long-term therapeutic intervention [6]. With the growing number of people with iSCI, there is an urgent need to target specific motor impairments in people with iSCI to maximize their motor recovery, including locomotion [6]. Studies have reported the possibility of regaining locomotor function even after many years of SCI [12,13], which could be achieved by increasing lower limb muscle strength [14]. Given that the strength of the knee extensors and hip flexors has been considered to be important for effective walking [14,15], this study hypothesized that strengthening these specific muscles could improve walking in people with iSCI. The recovery of muscle power after iSCI has a significant impact on how well an individual can walk functionally [16]. Additionally, the ability to execute daily activities (including standing, walking, climbing, etc.) needs adequate lower extremity muscle strength [14]. Therefore, further, research is needed to investigate the impact of specific muscle impairment following iSCI on locomotion ability [7] to explore the potential treatments that may enhance particularly affected muscle strength and improve walking ability in people with iSCI.

Conventional treatment typically prioritizes using the intact muscles to attain compensatory function. Given that there is little spontaneous progress on motor recovery [17], it is necessary to provide proper rehabilitation to improve functional mobility, muscle strength, and balance in people with iSCI [18]. Activity-based therapy (ABT) aims to engage muscles caudal to the level of injury so as to restore/re-educate the neuromuscular system and to regain a particular motor activity [5,19]. Therapies for chronic SCI may reduce long-term consequences and restore function loss [20]. Transcutaneous spinal cord electrical stimulation (TSCS) is an emerging treatment with promising outcomes to improve motor function beyond conventional therapy in people with chronic SCI [3]. It is a non-invasive method that uses superficial skin electrodes placed over the spinous processes [1,21]. TSCS could modulate post-SCI motor function [22]. It has been suggested that spinal neuromodulation, when combined with task-specific therapies, may produce better outcomes with long-term gains in functional recovery [18]. Although there is growing evidence that both TSCS and ABT are potential treatments for sensory and motor recovery in people with chronic SCI [18], the combined effects of both modalities on specific targeted muscles of the lower limb for improving walking ability remain unknown. 

TSCS has been used in conjunction with locomotor training, robotic-assisted training and treadmill training to improve gait and locomotion in people with iSCI [12,13,23]. It has been reported that the combined intervention of stimulation and task-based activity could produce better results [21]. The current study aimed to investigate the effects of augmenting ABT to TSCS treatment on enhancing specific lower limb muscle strength and improving locomotor ability in an individual with chronic iSCI. Since paralysis is associated with decreased muscle strength and impaired locomotion capacity [24], the findings of this study could benefit clinicians and rehabilitation professionals in planning specific locomotor rehabilitation programs for improving muscular force and walking function in this population. To the best of our knowledge, this is the first study to the compare effectiveness of ABT against ABT+TSCS in enhancing walking in people with chronic iSCI. 

## 2. Materials and Methods

The study was approved by the Human Subjects Ethics Sub-Committee of The Hong Kong Polytechnic University (Reference no: HSEARS20190201002-01) and written informed consent was obtained from the study participant.

### 2.1. Clinical Characteristics of the Participant

The participant was a 45-year-old male with chronic iSCI who experienced a traumatic thoracic cord injury at the T12 level in a road traffic accident, which occurred 16 years ago. According to the International Standards for Neurological Classification of Spinal Cord Injury (ISNCSCI) and American Spinal Injury Association Impairment Scale (AIS), the injury characteristic was graded as AIS-B. 

Most of his lower limb functions had been retained, and he was able to stand independently and walk a few steps with assistance. His lower limb muscles (hip flexors and knee extensors) were extremely weak, and he could maintain a standing balance for 5 min with great difficulty. In addition, he had a slight foot drop bilaterally, including spasticity of the lower extremity muscles, which impaired coordination and equilibrium. Both legs had observable voluntary movement and the muscle strength grading was between grades one and two.

### 2.2. Procedures

The treatment was administered in two phases, each lasting for 10 weeks, and assessments were conducted prior to intervention (pre) and after the end of each intervention (post). Phase 1 comprised ABT alone, and phase 2 included a combined ABT+TSCS. The participant received treatment two to three sessions per week, where each session lasted for approximately two hours. In total, the participant attended 22 sessions of ABT and 26 sessions of ABT+TSCS treatment in the current study. This treatment dosage was chosen because prior studies showed that clinical benefits, including increased muscular strength, might be achieved after 8–12 weeks of therapeutic interventions [25,26]. 

ABT tasks involved (i) passive manual stretching; (ii) isometrics, progressive resistance and active assisted exercises; (iii) weight-bearing balance training; (iv) locomotion training; and (v) motor-assisted cycling. Two experienced physiotherapists delivered the treatments and carried out overall assessments. 

The pre-assessments of muscle strength, walking ability, electromyographic response, and the spasticity of the lower limbs were conducted prior to the treatment session. The participant was not provided with any assistance during assessments but was continuously monitored by a physiotherapist to prevent falling. All the assessments were performed in the absence of stimulation. He was instructed to report any discomfort during stimulation, and the stimulation was turned off during the rest period. The rest (5 min) was provided in between the treatment sessions to avoid fatigue. The blood pressure and heart rate were measured every 15 min during training to monitor the cardiovascular response and to avoid episodes of autonomic dysreflexia. Additionally, at the end of each session, a skin inspection was conducted to observe signs of erythema and redness to rule out the possibility of any tissue damage. 

### 2.3. Experimental Protocol

In this study, T10-T11 and T12-L1 segments were stimulated using two specifically designed constant current stimulators (DS8R, Digitimer, Welwyn Garden City, UK) and a function generator (AFG1022, manufactured by Tektronix, Inc., Beaverton, OR, USA) that triggered the stimulators and produced a 10 kHz burst signal delivered at 30 Hz [13,27,28]. The stimulation parameters were set at the start of each session and were kept constant throughout the session. However, the intensities varied for each session. Based on the participant’s tolerance, it ranged from 105 mA to 130 mA. 

Previously, it was reported that a frequency of 30–50 Hz could produce therapeutic benefits and enhance gait function [29], while 30 Hz is optimal for eliciting a greater motor response [30]. We tested at 20 Hz, 25 Hz, and 30 Hz and established that, at 30 Hz, the participant felt comfortable and supported when executing the ABT tasks. In addition, he could tolerate greater intensities and experience better comfort and stability. Similarly, prior studies using a 10 kHz carrier frequency have shown to enhance locomotor function in people with iSCI when combined with exercise training [13,31]. 

A study revealed that stimulating T10-T11 (L1-L2 spinal cord segments) showed activation of anterior rectus femoris (RF) and vastus lateralis, and T12-L1 (L5-S1 spinal cord segments) activated hamstrings, triceps surae, and tibialis anterior (TA) muscles, respectively [29]. Likewise, stimulation at T11-12 elicited an increment in the quadriceps and hamstring muscle activity, while at L1-L2, it induced an elevation in the triceps surae and TA [30]. Additionally, the anterior RF and vastus lateralis showed a larger activity during T10-T11 stimulation, whereas the hamstrings and TA exhibited a stronger motor response during T12-L1 stimulation [30]. Based on these literatures, the above-mentioned stimulation site and parameters were used in this study. 

### 2.4. Outcome Measures

The primary outcome measures comprised following assessments: (1) manual muscle testing (MMT) to measure the strength of lower limb muscles, which was assessed on a range from 0–5 with a scale of five intervals [32]; (2) a 6 min walk test (6MWT) to assess changes in walking performance capacity; and (3) surface electromyography (EMG) (Model DE-2.1; Delsys USA, Inc., Boston, MA, USA) to measure muscle activities of lower limbs. Surface EMG electrodes were placed on anterior RF, hamstring (biceps femoris-BF), medial gastrocnemius (MG), and TA. The EMG response was recorded during a sit-to-stand, stand-to-sit, and squatting tasks with the support of a walker.

During the 6MWT, the participant was given 6 min to walk with a walker as far as he could in a gait lab (where 6 m had been marked) without stopping until absolutely necessary. He was allowed to take breaks as required throughout the test, but the timer did not pause, and he was expected to resume walking once he was physically fit to do so. Verbal feedback or words of encouragement were given after every one minute throughout the test. Later, the total distance reached in six minutes was documented.

Secondary outcome measures included the 11-point numeric pain-rating scale (NPRS), where 0 means no pain, while 10 means the worst imaginable pain [33]. Additionally, ISNCSCI was used to measure the sensory and motor changes, while the Modified Ashworth Scale (MAS) was used to measure the spasticity. 

### 2.5. Data Analysis

The data were graphically and statistically analyzed through GraphPad Prism version 9.0. The EMG signal was digitized at a 2 kHz sampling rate and was analyzed offline using customized written scripts in MATLAB (version 2016a, The MathWorks Inc., Natick, MA, USA). For each variable that was analyzed, descriptive statistics (mean ± SD) were calculated to observe changes in assessment scores. To examine how each treatment affected our participant’s outcome, separate Wilcoxon matched-pair signed-rank tests were used to compare the temporal changes of outcomes after each phase. The root mean square (RMS) value of the EMG of each muscle was calculated in millivolts (mV) and described as a mean ± standard deviation (SD) to compare the differences between two treatments (ABT and ABT+TSCS). For all statistical analyses, the level of significance was set at 0.05.

## 3. Results

**Figure 1:**
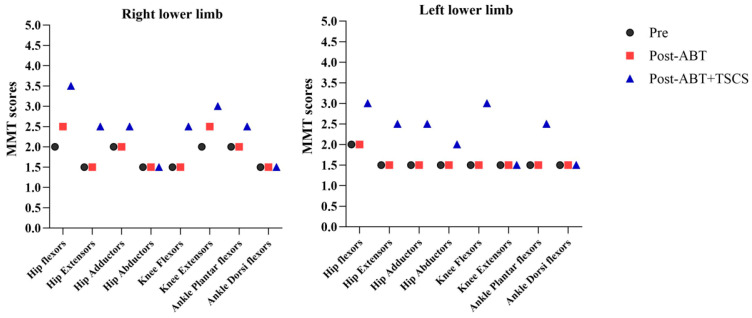
The manual muscle testing (MMT) scores measured from lower limbs in grades (0–5) during three different assessment periods (ABT: Activity-based Therapy; TSCS: Transcutaneous electrical spinal cord stimulation).

Of all the lower limb muscles tested, only the hip flexors (right: from 2/5 to 2.5/5), and knee extensors (right: from 2/5 to 2.5/5) had a slight increase in muscle strength grading after the ABT period (Figure 1). However, after the combined treatment (ABT+TSCS), the majority of lower limb muscles, i.e., hip flexors (right: from 2/5 to 3.5/5; left: from 2/5 to 3/5), hip extensors (right: from 1.5/5 to 2.5/5; left: from 1.5/5 to 2.5/5), hip adductors (right: from 2/5 to 2.5/5; left: from 1.5/5 to 2.5/5), hip abductors (left: from 1.5/5 to 2/5), knee flexors (right: from 1.5/5 to 2.5/5; left: from 1.5/to 3/5), knee extensors (right: from 2.5/5 to 3/5), and ankle plantar flexors (right: from 2/5 to 2.5/5; left: from 1.5/5 to 2.5/5) showed increases in muscle strength. The overall mean ± SD for MMT was 1.7 ± 0.2 at pre-intervention, which slightly changed to 1.8 ± 0.3 after post-ABT, and further increased to 2.2 ± 0.6 after post-ABT+TSCS. However, significant increases in MMT scores were noted after the ABT+TSCS treatment (*p* = 0.015), rather than after ABT alone (*p* = 0.250). 

**Figure 2:**
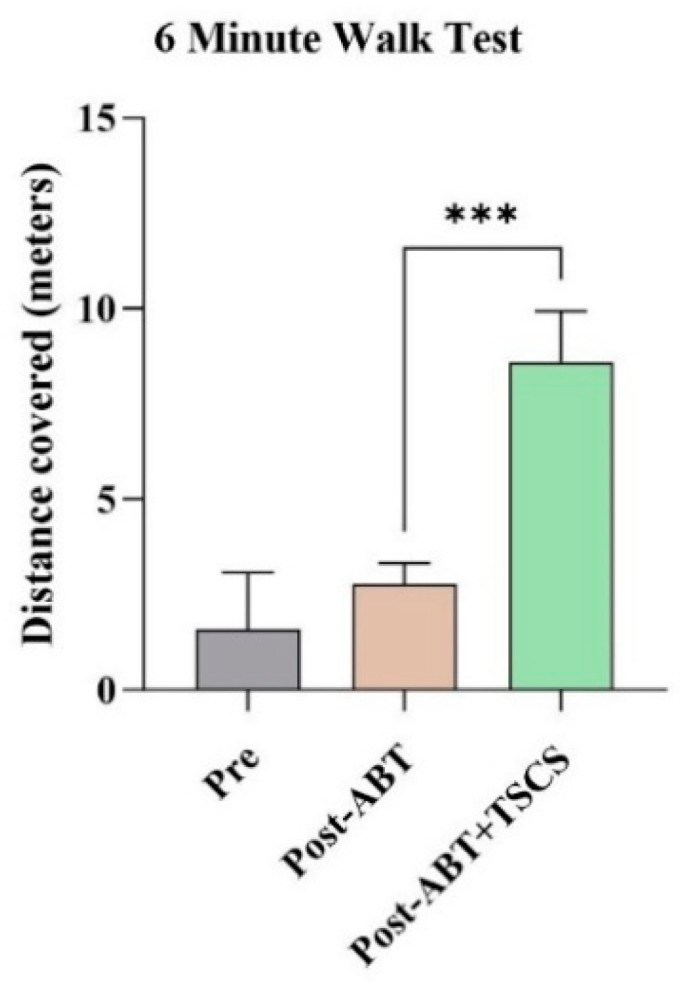
The distance covered by the participant during 6 min walk test in three distinct evaluation stages (ABT: activity-based therapy; TSCS: transcutaneous electrical spinal cord stimulation; *** *p* < 0.001).

The participant’s walking ability also improved. At pre-assessment, 6MWT showed that the participant struggled to complete the task and could only walk for three meters (m) with difficulty. Although 10 weeks of ABT treatment did not show significant progress in walking distance (3.5 m, *p* = 0.125), 6MWT walking distance increased to 10 m after ABT+TSCS (*p* = 0.031) (Figure 2). Additionally, balance and stability were improved after combined treatment than ABT alone. 

**Figure 3:**
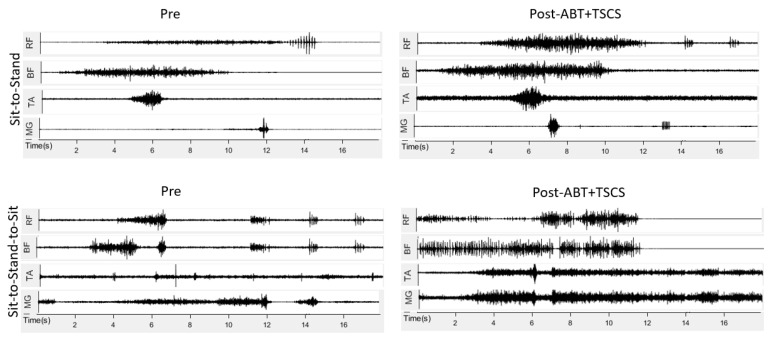
The electromyography (EMG) signals recorded from lower limb muscles in millivolts (mV) during three different assessment phases (ABT: activity-based therapy; TSCS: transcutaneous electrical spinal cord stimulation; RF: rectus femoris; BF: biceps femoris; MG: medial gastrocnemius; TA: tibialis anterior).

The EMG demonstrated an increase in the RMS value of lower limb muscles during sit-to-stand and sit-to-stand-to-sit tasks after ABT+TSCS, whereas there was no significant change in EMG signals after ABT alone. All lower limb muscles demonstrated increases in the EMG response following the combined treatment (Figure 3). The RF (right: from 0.02 ± 0.01 mV to 0.09 ± 0.02 mV; left: from 0.03 ± 0.01 mV to 0.07 ± 0.03 mV) and BF (right: from 0.05 ± 0.04 mV to 0.07 ± 0.05 mV; left; from 0.04 ± 0.02 mV to 0.06 ± 0.03 mV) showed a greater increase in EMG signals after ABT+TSCS treatment as compared to ABT alone. Similarly, the EMG values of MG and TA were also increased, (right: from 0.03 ± 0.01 mV to 0.05 ± 0.04 mV; left: from 0.02 ± 0.02 mV to 0.05 ± 0.03 mV) and (right: from 0.05 ± 0.04 mV to 0.07 ± 0.05 mV; left: from 0.03 ± 0.02 to 0.06 ± 0.05 mV), respectively, after ABT+TSCS treatment. The RF and BF showed greater activation (increased EMG signals) post-ABT+TSCS during the sit-to-stand task followed by increased EMG response of TA and MG, respectively. In addition, when the sit-to-stand-to-sit task was performed, the EMG signals recorded revealed higher RMS for RF, BF, TA, and MG post-ABT+TSCS than post-ABT.

**Figure 4:**
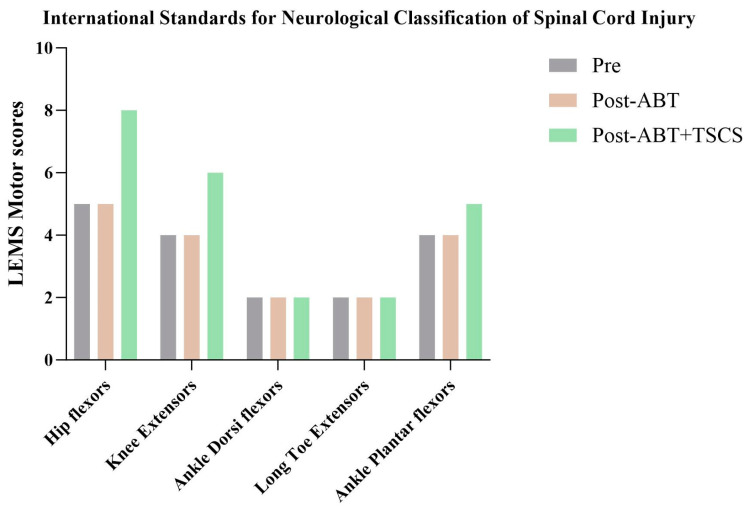
The lower limb motor scores (LEMS), as measured by International Standards for Neurological Classification of Spinal Cord Injury (ISNCSCI), during three distinct assessment stages (ABT: activity-based therapy; TSCS: transcutaneous electrical spinal cord stimulation).

The ISNCSCI (Figure 4) showed that the motor scores of lower limb muscles increased after receiving ABT+TSCS treatment. Additionally, the sensations were found to be normal across all timepoints and were, therefore, excluded from the analysis. The overall lower extremity motor score was 17 pre-intervention, which remained unchanged after ABT, but further increased to 23 points after ABT+TSCS treatment. Specifically, scores of hip flexors, knee extensors, and ankle plantar flexors increased by 3 points, 2 points and 1 point, respectively. 

**Figure 5:**
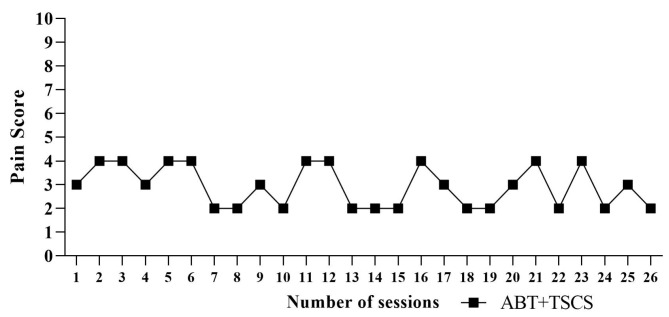
The pain ratings throughout ABT+TSCS sessions measured by an 11-point numeric pain rating scale (NPRS) (ABT: activity-based therapy; TSCS: transcutaneous electrical spinal cord stimulation).

The participant’s post-treatment NPRS varied between 2 and 4 (Figure 5). He had almost no or very little discomfort after the ABT+TSCS treatment. Additionally, there was no incidence of pain complaints that required the suspension of stimulation or adjustments of the stimulation settings. The participant experienced skin redness and dryness (5–6 episodes) after the combined treatment, but these side effects were modest and did not require medical attention. It disappeared in 3–4 days. 

**Figure 6:**
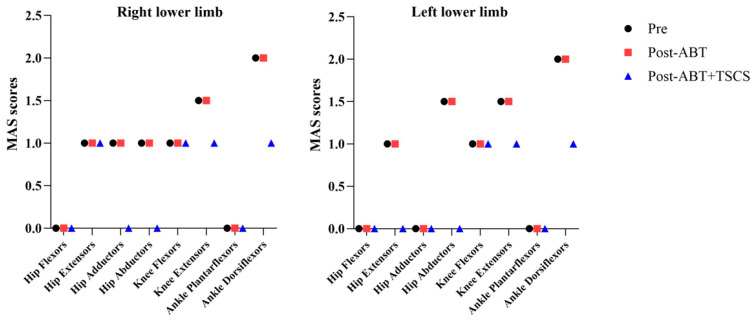
The five-point Modified Ashworth Scale (MAS) scores of lower limbs during three different measurement periods (ABT: activity-based therapy; TSCS: transcutaneous electrical spinal cord stimulation).

The MAS score of hip adductors (right: from 1/4 to 0/4), hip abductors (right: from 1/4 to 0/4; left: from 1.5/4 to 0/4), knee extensors (right: from 1.5/4 to 1/4; left; 1.5/4 to 1/4), and ankle dorsiflexors (right: from 2/4 to 1/4; left; 2/4 to 1/4) showed a reduction in spasticity after the ABT+TSCS treatment (*p* = 0.125). However, no absolute change in the spasticity was observed following ABT alone. Figure 6 shows that the overall mean ± SD for MAS pre-intervention was 0.9 ± 0.8, which remained almost unchanged (0.8 ± 0.7) post-ABT but further decreased to 0.5 ± 0.6 post-ABT+TSCS treatment. However, all these changes were not statistically significant.

## 4. Discussion

The current study examined the effects of ABT+TSCS on specific lower limb muscle strength and walking performance in an individual with iSCI. The findings support the idea that ABT+TSCS could be used as a potential treatment in people with chronic iSCI. In contrast, a previous study demonstrated improved lower limb function, including standing following combined stimulation and ABT, yet lacked a direct comparison between ABT and epidural stimulation [34]. Our earlier work with a transcutaneous approach has shown promise in restoring function post-SCI [1]. The findings of this study underscore the clinical value of TSCS, which is non-invasive, cost-effective, user-friendly, and associated with minimal side effects, as opposed to the more invasive epidural stimulation [1,34].

The restoration of muscle strength in people with iSCI can enhance functional skills, including locomotion. Prior research has shown that hip flexors [16] and ankle plantar flexors play a crucial role in locomotor function, which generate the majority of the propelling forces required for effective walking [6]. In the present study, the hip flexors strength (grade 3.5/5) was greatly increased, followed by hip extensors (grade 2.5/5), knee extensors (grade 3/5), and ankle plantar flexors (grade 2.5/5). It has been reported that the restoration of muscle strength more than grade 3 in the affected quadriceps (i.e., hip flexors) post-SCI was associated with a better prognosis of walking [15]. In addition, knee extensors with at least grade 3/5 strength are reported to be necessary for locomotion [16]. The hip flexors are essential for bringing the moving leg forward during the initial swing phase of walking [16], whereas hip extensors assist the balance during standing [16]. The elevated strengths of hip flexors and extensors could be the potential reason for our participant’s improved walking ability. TSCS may lower the activation threshold of spinal motor neurons and increases residual descending volleys during volitional movement [18], which facilitates the activation of previously weak or paralyzed muscles and allows people with SCI to more actively participate in training [18]. 

This study supports the use of TSCS for increasing muscle power and improving locomotion in individuals with iSCI. A prior case study reported that an individual with chronic SCI regained volitional control of paralyzed lower extremities and improved standing after 52 sessions of TSCS treatments with treadmill and motor-assisted cycling [13]. A pilot study combining TSCS with sit-to-stand training revealed the recovery of volitional control in people with complete and iSCI [35]. Likewise, another study evaluating combined TSCS and gait training found significant improvements in walking function in people with iSCI; however, it used a body weight support system for locomotion training [36], which requires expensive equipment and trained personnel for training. Conversely, our study focused on an individual’s voluntary effort for training that could provide self-confidence and reduce dependence on external support devices. While the main outcomes in previous studies were joint range of motion, EMG, and walking function [35,36], this study also evaluated MMT and NPRS. The MMT results showed an increase in the muscle strength of specific muscles which could be one reason for the improved locomotion. The lack of severe TSCS-related discomfort also supported the feasibility of using TSCS for people with iSCI, which was similar to prior research [37]. 

The present study found that ABT+TSCS was better than ABT alone in improving lower limb strength and ambulation ability. Notably, the current study reported that after ABT+TSCS treatment, 6MWT distance increased by threefold as compared to ABT alone, and the participant could walk a significantly longer distance with improved stability. A previous scoping review also found that, compared to ABT alone, TSCS combined with ABT could assist people with complete or incomplete SCI to have greater improvements in walking speed and ability to stand without assistance [18]. It is known that intensive ABT alone is insufficient to restore standing and walking in people with chronic motor full paralysis; these people need activating neuromodulatory factors [38]. The neuromodulatory action of TSCS improves motor function after spinal cord damage by activating local or propriospinal motor control mechanisms and potentiating descending corticospinal acting on spinal motoneurons [29]. Collectively, our results indicate that the stimulation of dorsal nerve roots may modulate interneuronal networks in the lumbosacral spinal cord [39] and reorganize spinal networks by intensive repeated practices when combined with TSCS, leading to long-term functional recovery [40].

A previous study reported that the EMG response of lower limb muscles in people with SCI was elevated during treadmill training, or robot-assisted and bipedal walking [13]. A study on healthy individuals using a single TSCS session demonstrated an immediate increase in the EMG response of quadriceps and TA [29]. Interestingly, when only TSCS was delivered to lower extremity muscles without any voluntary effort to take a step, no EMG activity was observed [41]. Our study showed that TSCS together with voluntary effort led to increased quadriceps (i.e., RF) and TA’s EMG activity during a sit-to-stand task, as well as an increase in muscle strength. Therefore, volitional motor function and EMG amplitude increased more greatly with the combined approach [18]. 

This study observed a slight decrease in spasticity after ABT+TSCS, while no change followed the ABT alone. This finding concurred with another study that found combining training with stimulation mitigated spasticity [31], although alterations in spasticity were not essential for a person with SCI to regain voluntary control over their paralyzed muscles [42]. 

## 5. Limitations and Recommendations

Despite the promising findings, this study had some limitations. Our results were obtained from a single participant, which might not be generalized to individuals with different levels or extents of SCI. Future studies should include a large sample size and participants with different SCI characteristics to validate our findings. In addition, future studies should include long-term follow-up to assess the lasting effects and sustainability of the treatment. Due to the inability to blind the physiotherapists to the treatments and assessments, there was a potential for bias. Additionally, the mechanism underlying the observed recovery is not fully understood. Further mechanistic research is required to understand these processes, enabling the development of individualized treatments with diverse injury profiles.

## 6. Conclusions

The findings from this study provide promising preliminary evidence that the combined treatment of ABT+TSCS may help people with chronic iSCI to regain or enhance their walking ability. The outcomes are essential because after only 26 sessions of ABT+TSCS, an individual with chronic iSCI had significant increases in target-specific muscle strength of lower limbs and locomotion ability. While it is reasonable to assume that the improvement may continue with a greater number of weekly sessions, future studies need to investigate the optimal dosage or duration of ABT+TSCS, as well as the carryover effect of treatment after the termination of treatments. 

## Data Availability

The raw data supporting the conclusions of this article will be made available by the authors on request. The data are not publicly available as we store our data in the database owned by the university, and they cannot be retrieved by others due to the data-sharing policy.
